# Migraine epidemiology, comorbidities and therapeutic landscape: a national population-based study

**DOI:** 10.3389/fneur.2026.1743203

**Published:** 2026-01-26

**Authors:** Nirit Lev, Lihie Sheffer, Ido Peles, Emily Elefant, Gal Ifergane

**Affiliations:** 1 Department of Neurology, Meir Medical Center, Clalit Health Services, Kfar Sava, Israel; 2 Gray Faculty of Medical and Health Sciences, Sagol School of Neuroscience, Tel Aviv University, Tel Aviv, Israel; 3 Clinical Research Center, Soroka University Medical Center and Faculty of Health Sciences, Ben Gurion University of the Negev, Beer Sheva, Israel; 4 Division of Brain Medicine, Department of Neurology, Soroka University Medical Center, Clalit Health Services, Beer Sheva, Israel

**Keywords:** comorbidities, epidemiology, migraine, prevalence, preventive treatment

## Abstract

**Background:**

Migraine is a leading cause of disability worldwide, yet national-level epidemiological data are often lacking, hindering public health planning. This study aimed to provide the first comprehensive, population-based assessment of migraine epidemiology, comorbidity burden, and preventive treatment patterns in Israel.

**Methods:**

We conducted a retrospective cohort study using electronic health records from Clalit Health Services (CHS), which insures over 50% of the Israeli population. 4,614,331 adults were included in the analysis. Patients with migraine were identified between 2000 and 2023 using physician-recorded ICD-9 codes or dispensed triptan prescription. Patients with migraine were matched 1:2 with non-migraine controls to assess comorbidities. We calculated point prevalence, annual incidence, and analyzed the preventive treatment landscape before and after the introduction of calcitonin gene related peptide (CGRP)-related migraine-specific preventive treatments.

**Results:**

The study included 356,441 patients with migraine and 4,257,890 controls. Migraine disproportionately affected females (75.8%) and younger adults (mean age 30.7 ± 13.2 years). Observed prevalence was lower than global estimates across most age strata. Incidence peaked among women aged 18–24 at 7.5 cases per 1,000 individuals. Patients with migraine carried a substantial comorbidity burden compared with age- and sex-matched controls. The highest adjusted odds ratios (ORs) were observed for chronic pain and psychiatric diseases (ORs for low back pain 2.67, fibromyalgia 2.42, endometriosis 1.86, anxiety 2.02, and depression 1.81). Vascular and metabolic conditions (hypertension, dyslipidemia, atrial fibrillation, and cerebrovascular disease) were more frequent, and stroke risk was significantly elevated. A negative association was found with diabetes. The proportion of patients who used preventive medication was low (9.6 and 8.8%, in 2018 and 2022 respectively) and did not increase after the introduction of migraine-specific treatments. Preventive use was most common in young adults (18–24 age group) and middle-aged adults (45–54 age group).

**Conclusion:**

This large national population-based study reveals a high comorbidity burden among patients with migraine and suggests significant underdiagnosis compared to global benchmarks. The use of preventive treatment remained strikingly low, including novel migraine-specific therapies. These findings underscore the need for improved migraine recognition, integrated multidisciplinary care, and policy-level strategies to reduce the burden of this disabling condition.

## Introduction

Migraine is one of the most disabling neurological disorders worldwide, ranking among the leading causes of years lived with disability (YLDs). Migraine significantly impairs quality of life and functional capacity during and between episodes. According to the Global Burden of Disease (GBD) study from 2021, migraine is the third-ranked condition contributing to nervous system disability-adjusted life years (DALYs), affecting an estimated 1.16 billion individuals globally (1). The GBD 2023 Headache Collaborators reported that in 2023, 2.9 billion individuals worldwide were affected by headache disorders, corresponding to a global age-standardized prevalence of 35% and a YLD rate of 542 per 100,000 population (2). Although tension-type headache was nearly twice as prevalent as migraine, migraine accounted for approximately 90% of YLDs attributed to headache disorders in 2023 (2).

In terms of age-standardized YLD rates across all conditions, migraine ranked eighth globally, with a rate of 487 YLDs per 100,000 population (2). The burden of headache disorders was approximately twice as high among females, reflecting both higher migraine prevalence and a greater proportion of time lived with headache compared with males (2). Moreover, the global burden of migraine among women of childbearing age has increased substantially over the past three decades (3). Beyond its direct clinical impact, migraine imposes substantial negative effects on families, workplaces, healthcare systems, and society as a whole (4).

Despite its substantial individual and societal burden, migraine remains underdiagnosed, undertreated, and often marginalized in health policy discussions compared to other chronic diseases. Understanding its epidemiology at a national level is critical for informed public health planning, resource allocation, and the development of targeted interventions.

Global initiatives, such as the GBD and Eurolight projects, have provided important prevalence estimates (1–7). However, national, population-based studies are scarce. Such data are essential because local demographic, cultural and health system factors shape diagnosis, comorbidities, and treatment patterns. In Israel, universal health coverage and centralized electronic medical records (EMRs) create an ideal setting for a comprehensive assessment. Yet, beyond regional studies (8), no national data has characterized the epidemiology, comorbidity burden, or treatment landscape of migraine.

Recently, therapeutic options have expanded with the advent of migraine-specific therapies, calcitonin gene-related peptide (CGRP) monoclonal antibodies and gepants. These highly effective agents raise new clinical and policy considerations, but real-world data on their use remains limited. We therefore conducted an extensive population-based study of adults insured by Clalit Health Services (CHS), which covers more than half of Israel’s population, to estimate migraine prevalence and incidence, evaluate comorbidities, and describe the utilization of preventive treatments.

## Methods

### Data source and setting

We conducted a population-based, retrospective cohort study using the EMR of CHS, Israel’s largest health maintenance organization. Israel’s healthcare system is a government-regulated national health insurance model, ensuring all residents are insured by one of four non-profit Health Maintenance Organizations (HMOs). CHS insures over 5.4 million individuals, representing more than half of Israel’s population and approximately two-thirds of the adult population. The CHS database integrates longitudinal data across primary and specialty care, hospitalizations, imaging, laboratories, and dispensed prescriptions, ensuring comprehensive follow-up of enrolled members (8–10).

### Study population and case definition

We included adults aged 18 years or older diagnosed with migraine between January 1st, 2000, and August 31st, 2023. Migraine was defined using two validated criteria: a physician-recorded diagnosis of migraine according to the International Classification of Diseases, 9th Revision (ICD-9), with or without aura, and/or at least one dispensed prescription for a triptan medication (ATC N02CC01–N02CC07).

Both general practitioners (GPs) and neurologists contribute to the diagnoses in CHS. The diagnosis of migraine in Israel is based on International Classification of Headache Disorders (ICHD-3) criteria, however similarly to other real-world EMR studies (11–15) the database is built on real-world clinically driven diagnosis. Overall, 70.3% of patients with a physician-recorded migraine diagnosis were also prescribed triptans during follow-up, indicating substantial concordance between diagnostic coding and migraine-specific treatment. Prior validation studies have demonstrated the high reliability of migraine coding against expert adjudication (16, 17). Individuals identified only through triptan dispensing were considered probable patients with migraine (8, 18, 19), as off-label triptan use (e.g., cluster headache) is rare in Israel.

### Control group and comorbidity ascertainment

Each patient with migraine was matched with two controls without migraine, based on age and sex. Comorbidities were identified using diagnostic codes recorded in CHS at any time before or during follow-up. Comorbidities were defined as the active diagnoses associated to the population in the EMR during the analyzed period. Relevant groups of comorbidities were selected from prior literature (13). They included: psychiatric disorders, cardiovascular, neurologic, digestive, pain, respiratory, rheumatologic, and metabolic conditions. The detailed list of analyzed comorbidities, along with the respective ICD-9 codes, is detailed in Supplementary Table S2.

### Primary and secondary outcomes

Point prevalence of migraine was calculated as of August 31st, 2023, using the adult CHS-insured population as the denominator. Indirect age-standardization was performed by 5-year strata against published global reference prevalence estimates.

Annual incidence was assessed for 2022. Incident cases were defined as adults with a first migraine diagnosis or triptan prescription in that year, with no prior history in the database. Rates were expressed per 1,000 individuals at risk, stratified by age and sex. Sex-specific analyses were performed given the well-established female predominance of migraine and documented sex differences in migraine epidemiology and disease burden (3, 20, 21).

Preventive treatment utilization; we identified dispensing of guideline-listed preventive drugs after migraine diagnosis, using Anatomical Therapeutic Chemical (ATC) codes. Classes included tricyclic antidepressants, serotonin–norepinephrine reuptake inhibitors (SNRIs), beta-blockers, angiotensin receptor blockers, anticonvulsants, and migraine-specific preventives (CGRP monoclonal antibodies and gepants). Onabotulinum toxin A prescriptions were not captured in the CHS database.

A complete drug–ATC code list is provided in Supplementary Table 1. Ever use was defined as ≥1 dispensing of any preventive drug at any time after diagnosis. Active use each year was defined as ≥1 dispensing in that calendar year, categorized by first active class.

### Statistical analysis

Descriptive statistics were used to summarize participant characteristics. Normally distributed continuous variables were summarized using means and standard deviations (SD), while non-normally distributed variables were reported as medians and interquartile ranges (IQR). Categorical variables were expressed as frequencies and percentages. Between-group comparisons (patients with migraine vs. controls) were conducted using *t*-tests, Mann–Whitney U tests, chi-square tests, or Fisher’s exact tests, as appropriate.

To compare migraine diagnosis rates in Israel with published global prevalence benchmarks, we performed indirect age standardization. The study population was divided into 5-year age groups. For each age group, we recorded the number of patients diagnosed with migraine as of August 31st, 2023, the total number of insured residents in that age group, the observed point prevalence per 10,000 adults, and the corresponding age-specific reference prevalence per 10,000 adults based on published global estimates. We then calculated the expected number of migraine cases by multiplying the reference prevalence by the number of individuals in each age group. This provided the number of migraine cases we would expect if our study population had the same age-specific prevalence profile as the reference population.

We calculated the annual incidence of migraine per 1,000 adults in 2022 by dividing the number of new migraine cases by the at-risk population. Incidence was also estimated separately by 5-year age groups and by sex. The at-risk population included all patients without any prior documented migraine diagnosis (ICD-9, with or without aura) and without any previous claim for triptan medications.

To compare comorbidity prevalence between the migraine and non-migraine groups, we applied two complementary analytical approaches. First, we performed a 1:2 matching of patients with migraine to controls based on age and sex, followed by univariate comparisons for each comorbidity. Second, we fitted multivariable binary logistic regression models to examine the association between migraine status and each comorbid condition, adjusting for age and sex. Results are reported as odds ratios (ORs) with 95% confidence intervals (CIs) and *p* values.

Preventive treatment patterns in patients diagnosed by 2018 and by 2022 were compared cross-sectionally, with χ^2^ tests assessing differences in ever use and active class distributions.

Given the large sample size, standardized mean differences (SMDs) were used to assess group differences as sample size–independent measures of effect magnitude. Analyses of preventive treatment utilization across calendar years were descriptive and interpreted using effect sizes rather than statistical significance testing. All analyses were performed using R version 4.4.1 (R Foundation for Statistical Computing, Vienna, Austria) in the RStudio 2024.04.2 environment (Posit PBC, Boston, MA, USA).

## Results

The study included 4,614,331 adults insured by CHS. Of these, 356,441 were identified as patients with migraine and 4,257,890 without migraine (Table 1). Patients with migraine were younger (mean age 30.7 ± 13.2 years vs. 36.3 ± 19.3 years, SMD = 0.34) and predominantly female (75.8% vs. 48.8%, SMD = 0.58). Age distribution differed significantly between the groups. Patients with migraine were more likely to belong to higher socioeconomic score categories (34.4% vs. 29.6%; SMD = 0.11). Ethnic distribution was also slightly different between patients with and without migraine (Table 1).

**Table 1:** Demographics and clinical characteristics.

Characteristics	Patients without migraine	Patients with migraine	SMD
	(*N* = 4,257,890)	(*N* = 356,441)	
Age, Mean (SD)	36.34 (19.30)	30.72 (13.21)	0.34
Age group, *N* (%)			0.11
18–24	1,896,348 (44.5%)	175,184 (49.1%)	
25–34	609,771 (14.3%)	70,082 (19.7%)	
35–44	484,884 (11.4%)	53,683 (15.1%)	
45–54	459,865 (10.8%)	36,507 (10.2%)	
55–64	306,843 (7.2%)	13,221 (3.7%)	
65–74	278,089 (6.5%)	5,810 (1.6%)	
75–84	166,232 (3.9%)	1,712 (0.5%)	
>84	56,038 (1.3%)	242 (0.1%)	
Sex, female, %	2,079,818 (48.8%)	270,257 (75.8%)	0.58
Ethnicity, Jewish, %	3,358,214 (78.9%)	280,012 (78.6%)	0.01
Socioeconomic score, *N* (%)			0.11
Very high	310,203 (7.3%)	29,467 (8.3%)	
High	949,322 (22.3%)	93,060 (26.1%)	
Medium	1,286,402 (30.2%)	107,437 (30.1%)	
Low	1,162,888 (27.3%)	86,614 (24.3%)	
Very low	298,831 (7.0%)	18,997 (5.3%)	

**Figure 1:**
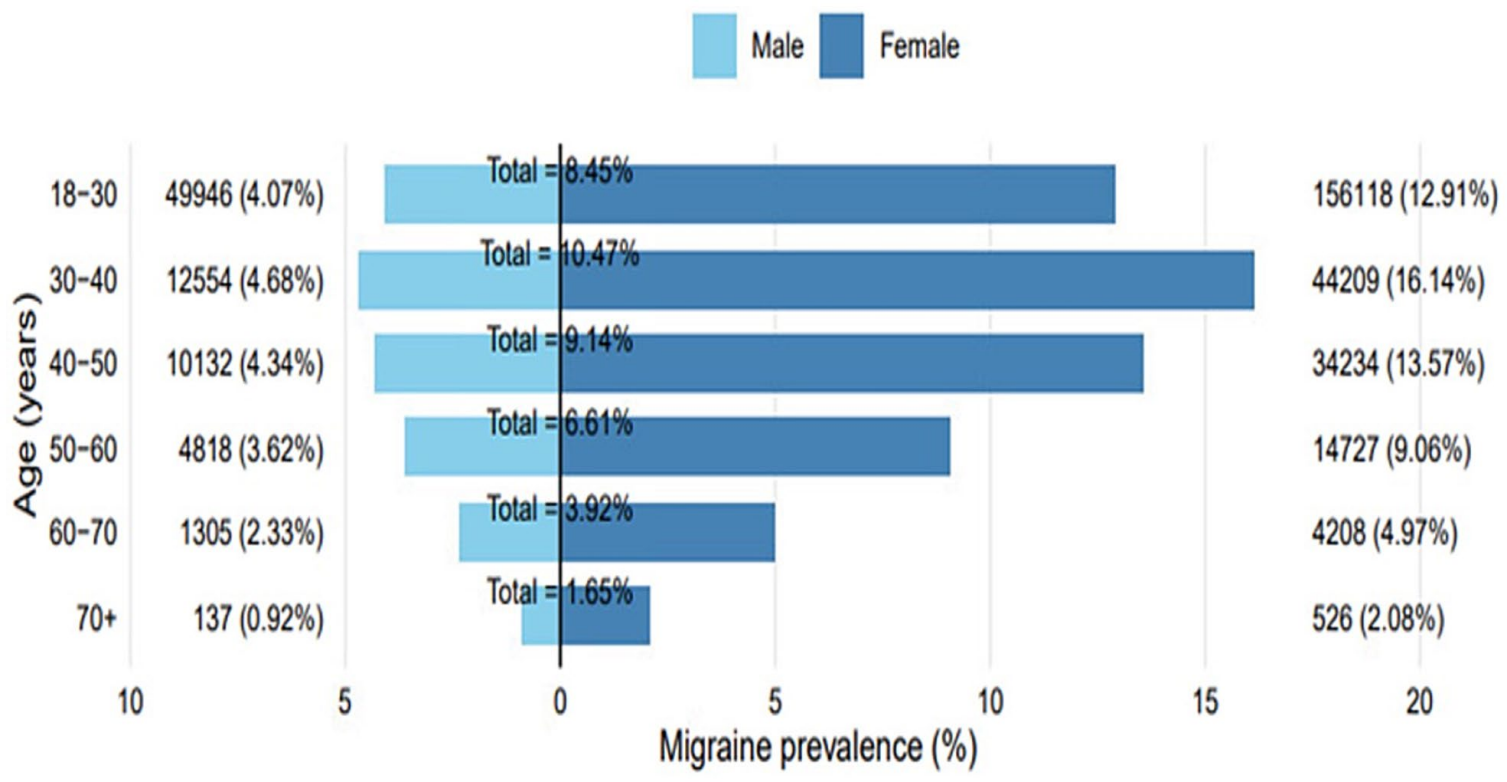
Age-to-sex pyramid of migraine prevalence. Standard age pyramid of migraine prevalence in Israel, highlighting female predominance and decline after age 50. Complements the prevalence data described in the results.

**Figure 2:**
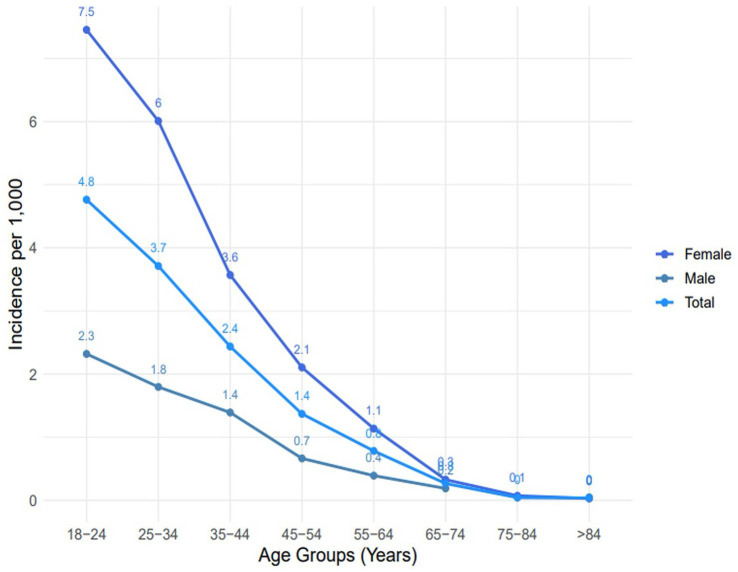
Annual migraine incidence per 1,000 people (2022). Age-specific incidence rates of new migraine diagnoses in 2022, expressed per 1,000 adults, stratified by the stated age intervals. Incidence peaked in the 18–24 age group and declined progressively with increasing age. Female incidence was consistently higher across all age groups.

Age-pyramid visualization highlighted female predominance and a marked decline in diagnoses after midlife (Figure 1). Distributions of age at migraine diagnosis, visualized using kernel density estimation, showed that women were diagnosed at a younger age on average, with peak incidence in the 18–24-year group (Supplementary Figure 1). The observed point prevalence of migraine in the CHS-insured adult population was lower than expected based on global reference rates across nearly all age groups. Prevalence peaked in the 30–39-year group at 10 per 1,000, whereas international estimates suggest a prevalence approximately double that rate. Detailed observed versus expected prevalence values are provided in Table 2. Annual incidence rates calculated for 2022 showed a sharp female predominance, peaking at 7.5 cases per 1,000 among women aged 18–24 compared with 2.3 per 1,000 in men of the same age group (Figure 2). Across all age groups, incidence rates were higher in women compared to men. Incidence declined steadily with advancing age in both sexes.

**Table 2:** Observed prevalence in Israel vs. world prevalence.

Age group	Observed patients	Total patients	Observed prevalence	Expected prevalence
18–19	72,574	1,017,204	7.13	17.58
20–24	102,033	1,039,811	9.81	19.24
25–29	38,897	379,583	10.2	20.29
30–34	30,447	285,364	10.7	21.14
35–39	27,351	255,609	10.7	21.41
40–44	24,707	247,407	9.99	21.56
45–49	20,088	235,579	8.53	20.07
50–54	13,423	182,103	7.37	18.23
55–59	6,188	109,361	5.66	16.39
60–64	3,747	85,732	4.37	14.08
65–69	1,621	48,566	3.34	11.74
70–74	482	21,789	2.21	9.47
75–79	110	8,588	1.28	7.93
80–84	14	3,827	0.366	6.89
85–89	9	2074	0.434	6.08
90–94	0	789	0	5
95+	0	329	0	3.7

**Figure 3:**
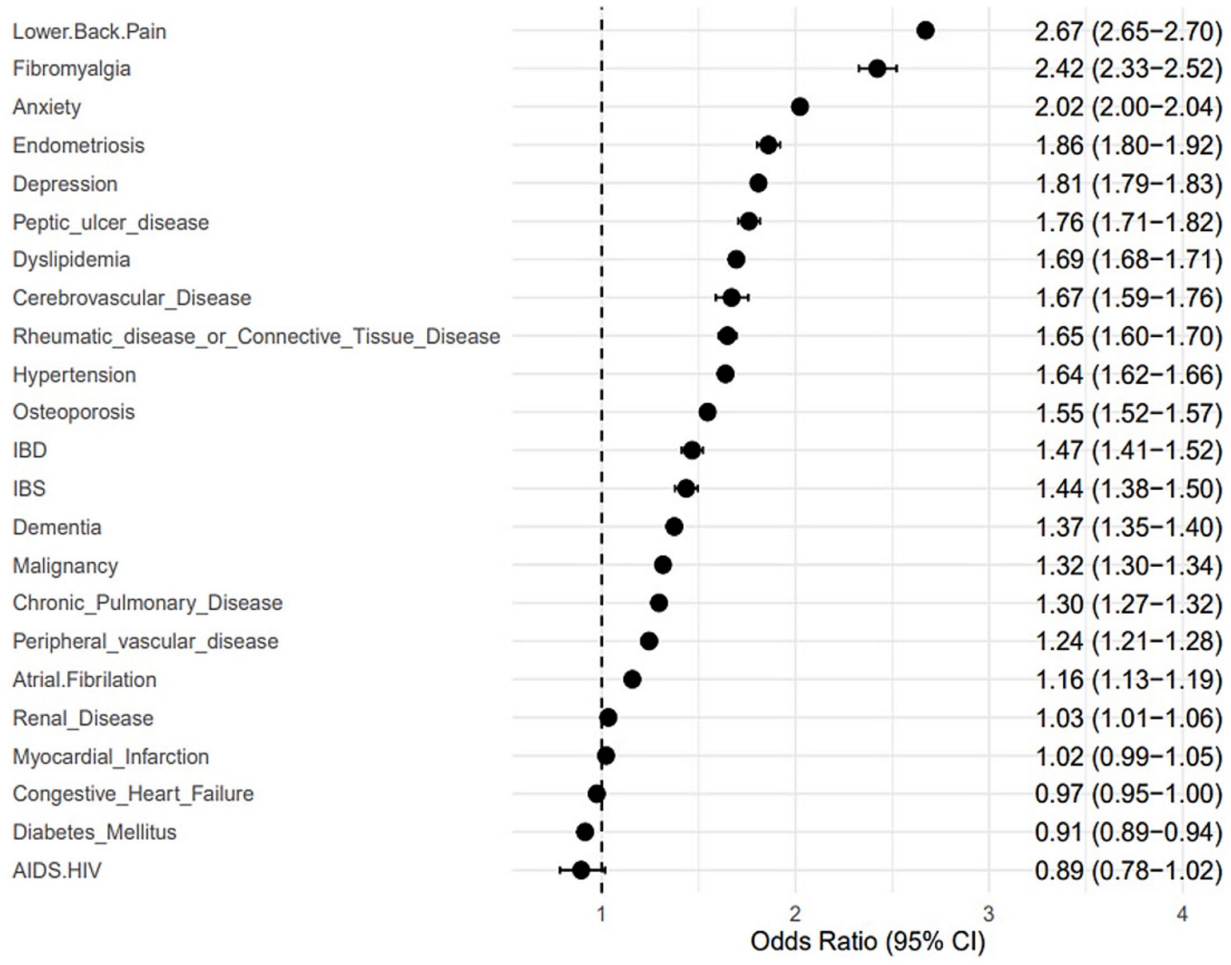
Relative odds of migraine (and 95% CI) vs. non-migraine controls for each comorbid condition. Relative odd ratios (ORs) of migraine and 95% confidence intervals (CIs) versus non-migraine matched controls were calculated for each comorbidity. Multivariable logistic regression models, adjusted for age and sex, were used to estimate the ORs and 95% CIs.

Patients with migraine carried a substantial comorbidity burden compared with age- and sex-matched controls. Psychiatric disorders were prevalent: anxiety (24.6% vs. 14.1%) and depression (18.6% vs. 11.5%) were both significantly elevated, corresponding to OR of 2.02 and 1.81, respectively. Chronic pain syndromes were also strikingly overrepresented. Low back pain was reported by 78.7% of patients with migraine compared with 58.4% of controls (OR 2.67), and fibromyalgia by 1.5% vs. 0.6% (OR 2.42). Endometriosis, a visceral pain disorder, was almost twice as common among patients with migraine (2.1% vs. 1.1%, OR 1.86). Vascular and metabolic conditions showed consistent associations. Hypertension (25.2% vs. 19.6%, OR 1.64) and dyslipidemia (36.1% vs. 27.7%, OR 1.69) were more prevalent among patients with migraine. The odds of cerebrovascular disease were also higher (0.8% vs. 0.5%, OR 1.67). Notably, diabetes mellitus was less common among patients with migraine than controls (1.9% vs. 2.1%, OR 0.91), an inverse association consistent with some previous epidemiologic reports (Table 3; Figure 3).

**Table 3:** Comorbidities with matched controls.

Diseases	Control group	Patients with migraine	SMD
	(*N* = 712,882)	(*N* = 356,441)	
Psychiatric diseases			
Anxiety, *N* (%)	100,588 (14.1%)	87,667 (24.6%)	0.28
Depression, *N* (%)	82,038 (11.5%)	66,156 (18.6%)	0.22
Chronic pain disorders			
Lower Back Pain, *N* (%)	416,276 (58.4%)	280,611 (78.7%)	0.45
Fibromyalgia, *N* (%)	4,431 (0.6%)	5,280 (1.5%)	0.09
Endometriosis, *N* (%)	8,109 (1.1%)	7,439 (2.1%)	0.08
Peptic Ulcer Disease, *N* (%)	8,482 (1.2%)	7,284 (2%)	0.07
Irritable Bowel Syndrome, *N* (%)	5,667 (0.8%)	3,969 (1.1%)	0.03
Vascular and metabolic diseases			
Hypertension, *N* (%)	139,820 (19.6%)	89,889 (25.2%)	0.14
Dyslipidemia, *N* (%)	197,117 (27.7%)	128,847 (36.1%)	0.18
Cerebrovascular disease, *N* (%)	3,493 (0.5%)	2,824 (0.8%)	0.04
Atrial Fibrillation, *N* (%)	16,140 (2.3%)	8,969 (2.5%)	0.01
Peripheral vascular disease, *N* (%)	11,724 (1.6%)	7,117 (2%)	0.04
Congestive heart failure, *N* (%)	17,165 (2.4%)	8,173 (2.3%)	0.01
Diabetes mellitus, *N* (%)	15,253 (2.1%)	6,883 (1.9%)	0.02
Osteoporosis, *N* (%)	53,447 (7.5%)	35,714 (10%)	0.09
Inflammatory diseases			
IBD, *N* (%)	6,656 (0.9%)	4,857 (1.4%)	0.05
Connective tissue diseases, *N* (%)	11,174 (1.6%)	8,906 (2.5%)	0.05
Other			
Renal diseases, *N* (%)	29,095 (4.1%)	14,682 (4.1%)	0.00
Chronic pulmonary disease, *N* (%)	36,280 (5.1%)	22,524 (6.3%)	0.05
AIDS/HIV, *N* (%)	739 (0.1%)	331 (0.1%)	0.00
Dementia, *N* (%)	25,153 (3.5%)	16,451 (4.6%)	0.06
Malignancy, *N* (%)	55,053 (7.7%)	34,038 (9.8%)	0.06

Preventive treatment use among patients with migraine was strikingly low. Among patients diagnosed by 2018, 9.6% had ever used prophylactic medication, compared with 8.8% in those diagnosed by 2022 (Table 4). Active use each year was lower still: 2.2% in 2018 and 2.5% in 2022. The most common preventive classes were tricyclic antidepressants and SNRIs (each 0.5–0.6% of patients), followed by beta-blockers (0.4–0.5%). Use of angiotensin receptor blockers doubled from 0.2 to 0.4% between 2018 and 2022. Anticonvulsant use declined slightly. Migraine-specific preventives were rare: by 2022, CGRP monoclonal antibodies were used by 0.1% of patients, and gepants were used by only few individuals.

**Table 4:** Preventive treatment landscape among patients with migraine diagnosed by 2018 and 2022.

Preventive treatment	2018 cohort	2022 cohort	Relative change %	SMD
Ever used preventive treatment, *N* (%)	27,223 (9.6%)	29,278 (8.8%)		0.03
Active preventive class				
Total, *N* (%)	6,507 (2.2%)	8,758 (2.5%)		
Tricyclic antidepressants, *N* (%)	1,619 (0.6%)	1,899 (0.5%)	−0.7%	
SNRI, *N* (%)	1,677 (0.6%)	2,243 (0.6%)	+13.2%	
Beta-Blockers, *N* (%)	1,500 (0.5%)	1,477 (0.4%)	−16.7%	
Angiotensin Receptor Blockers, *N* (%)	479 (0.2%)	1,309 (0.4%)	+131.2%	
Anticonvulsant, *N* (%)	737 (0.3%)	810 (0.2%)	−7.0%	
CGRP Monoclonal Antibodies, *N* (%)*	0 (0.0%)	242 (0.1%)		
Gepants, *N* (%)*	0 (0.0%)	3 (0%)		
Combined, *N* (%)	495 (0.2%)	775 (0.2%)	+32.5%	
Active preventive treatment by age group				
18–24, *N* (%)	1,564 (1.19%)	2,459 (1.46%)	+23%	
25–34, *N* (%)	1,088 (1.86%)	1,609 (2.37%)	+27%	
35–44, *N* (%)	1,384 (2.94%)	1,763 (3.42%)	+16%	
45–54, *N* (%)	1,559 (4.87%)	1,986 (5.91%)	+21%	
55–64, *N* (%)	665 (6.31%)	775 (7.46%)	+18%	
65–74, *N* (%)	229 (6.86%)	160 (6.35%)	−7%	
75+, *N* (%)	18 (4.46%)	6 (3.16%)	−29%	

*Classes first marketed post-2018 (e.g., CGRP mAb, gepants). Combined, concurrent treatment with multi-class preventive drugs.

Among the patients who took preventive treatments, most were young adults or middle-aged adults (24–28% of users in the 18–24 age group, and 22–24% in the 45–54 group, in 2018 and 2022 respectively). Preventive treatment use was nearly absent in those aged 65 years or older. Comparing active preventive use between 2022 and 2018, demonstrated increased use in younger age groups, with the highest increase in the 25–34 age strata (prophylaxis used increased by 27%), while there was a decrease in older age groups (by 7% in 65–74 and by 29% in patients older than 75) (Table 4).

## Discussion

In this large population-based study, we analyzed the demographic and clinical characteristics of 356,441 adult patients with migraine as compared to 4,257,890 patients without migraine within the CHS database, representing more than half of the Israeli population. Our findings provide the most comprehensive national data to date on the epidemiology, comorbidity burden and therapeutic landscape of migraine in Israel.

Several key insights emerged. As in other countries, migraine disproportionately affects women and younger adults, yet observed prevalence was consistently lower than global benchmarks, suggesting underdiagnosis. Patients with migraine carried a high burden of psychiatric, chronic pain, and vascular comorbidities. The use of preventive treatment remained low, with strikingly low uptake of novel migraine-specific therapies.

The age and sex distribution of migraine in Israel mirrors international patterns, with a threefold female predominance and peak incidence in early adulthood (21–23). However, prevalence rates across all strata were significantly lower than global estimates, indicating substantial underdiagnosis. It is important to note that the prevalence estimates reported in this study reflect clinically diagnosed migraine captured in EMR, rather than population-based prevalence derived from standardized diagnostic interviews. Accordingly, these estimates represent recognized and treated migraine and are expected to underestimate the true population prevalence.

Migraine prevalence peaked in the third and fourth decades of life and declined thereafter, mirroring the age-related pattern observed in other high-income countries (21, 23). However, compared to expected prevalence rates based on international benchmarks, the observed age-specific prevalence in our population was markedly lower across most strata. These findings suggest potential underdiagnosis or underreporting of migraine in Israel, particularly among younger individuals. This discrepancy may reflect both patient- and system-level barriers. Contributing factors likely include limited awareness (24), stigma, diagnostic variability in primary care (4), and restricted access to specialized headache services (8, 25–27). These barriers are particularly notable given Israel’s universal health coverage and integrated electronic health records, where economic access should not represent a limiting factor.

The design of this study enabled the examination of migraine comorbidities in a large, valid clinical database. Patients with migraine demonstrated elevated rates of psychiatric, chronic pain, and vascular conditions compared to matched controls. These findings are consistent with bidirectional associations between migraine and psychiatric disorders observed globally (27–31). Shared alterations in serotonergic, hypothalamic–pituitary–adrenal axis, neuropeptides (such as CGRP, pituitary adenylate cyclase-activating polypeptide (PACAP), substance P, neuropeptide Y and orexins) and genetic mechanisms may contribute to this phenomenon (32–34).

Significantly elevated rates of chronic pain disorders were found. Musculoskeletal pain disorders, particularly lower back pain and fibromyalgia were more prevalent in patients with migraine. Additionally, patients with migraine were more likely to report visceral pain conditions, such as endometriosis, inflammatory bowel disease (IBD) and peptic disease, echoing findings from previous studies (35–41). These associations reinforce the overlap with multisystem pain syndromes, emphasizing the need for multidisciplinary management approaches for patients with overlapping pain syndromes.

Hypertension, dyslipidemia, atrial fibrillation, and cerebrovascular disease were more frequent, and stroke risk was significantly elevated, aligning with previous studies and meta-analyses linking migraine to vascular outcomes (42–56). An increased risk of hypertension in patients with migraine was previously reported by several studies from different geographic areas (42–46) but was not found by all the studies (47). Similarly, most of the studies who investigated the association between migraine and dyslipidemia found a positive association between the conditions (42, 44, 46, 48). Association of migraine and atrial fibrillation was also supported by previous studies (49–51), including two large high-quality prospective population-based studies (50, 51), which showed a significant association with migraine with aura.

We found that patients with migraine have increased risk for stroke (OR 1.67). This finding aligns with previous studies (44, 52–55, 57), including a large meta-analysis (55). The interplay between migraine and stroke is complex, involving shared pathophysiology and overlapping risk factors (56). While migraine can serve as both a cause and a risk factor for stroke, the precise mechanisms remain unclear.

Negative association was found with diabetes. The association of migraine and diabetes was examined by several studies (42, 58–62) with mixed results. Negative association was reported by several previous studies (60–62). Further studies are needed to clarify this unexplained finding. These patterns underline migraine as part of a broader systemic vulnerability rather than an isolated neurological disorder.

Despite the disease burden, fewer than 10% of diagnosed patients had ever received preventive therapy, with minimal change after the introduction of CGRP monoclonal antibodies. Preventive use was concentrated in younger and middle-aged adults and was nearly absent in older groups.

Previous studies report that although around a third of patients with migraine would benefit from preventive treatment, globally only 1.6–19% use such drugs, and adherence has been found to be low (22, 63). Cross-sectional surveys found different prophylaxis use in different countries: in Europe around 15% (with a wide range between different countries) (64), in the USA 13–16.8% (65), in Japan 9.2–10.2% (66, 67). Despite high consultation and diagnosis rates among Polish patients with migraine, only 11.49% were treated by preventive medications (27, 68). Population-based data on utilization of prophylactic therapies are sparse. A Danish population-based health databases study reported prophylactic treatment was used by only 7% and that non-persistence with initial prophylactic treatment was high (69). A Swedish registry-based cross-sectional study found that 12% use of preventive migraine medications concomitantly with triptans (70).

Israel’s healthcare system operates under a government-regulated national health insurance model, ensuring universal coverage through four non-profit HMOs, of which CHS is the largest. Migraine care is primarily delivered in the community, with most patients initially managed by primary care physicians (GPs). Referral to neurologists is common but not mandatory, as patients may also access community neurologists directly without formal referral. Specialized headache care is provided mainly in dedicated outpatient headache clinics, which typically require referral and are characterized by long waiting times within the public system. As a result, some patients seek private neurological consultations, particularly for complex or refractory headache disorders. Similar patterns have been reported in other countries with universal healthcare systems, suggesting that coverage alone does not ensure timely or equitable access to specialist headache care (27, 68, 71). Accordingly, although financial barriers are unlikely to be the primary driver of low preventive treatment utilization in Israel, the observed gap likely reflects a combination of diagnostic inertia in primary care, limited access to headache specialists, physician and patient preferences, disparities in access, particularly in peripheral regions, and suboptimal adherence. These factors are consistent with international reports demonstrating persistently low use of migraine prophylaxis despite clear indications (22, 26, 63–67, 71).

Our findings highlight urgent gaps in migraine care. Underdiagnosis delays effective treatment and prolongs disability. Given the high comorbidity burden identified, migraine should warrant comprehensive assessment and integrated management. Interventions should include targeted education for primary care physicians, decision-support tools within EMRs to improve diagnostic accuracy and recommendations for preventive treatment candidacy, and structured referral pathways.

Population-level interventions, including digital outreach and public awareness campaigns may improve recognition. There is a need to educate not only patients and doctors, but also the general public, pharmacists, pharmaceutical companies, and the media in the context of migraine as a neurological disease causing severe disability in order to reduce stigma and increase awareness. At the policy level, migraine should be integrated into national chronic disease frameworks, and the cost-effectiveness of wider preventive use, including CGRP therapies, should be evaluated in a system where affordability is not the primary obstacle.

Key strengths of this study include the large, representative cohort, comprehensive EMR data, and study within a universal coverage system, minimizing selection bias. The CHS database captures a wide spectrum of sociodemographic and clinical information, allowing robust adjustment and subgroup analyses. The structure of the health care delivery system that provides universal access to primary clinics, expert consultations, and hospital care, combined with complete availability of EMRs and prescriptions at all levels of care, gives a unique opportunity to study the epidemiology of clinically validated migraine in a large population.

Limitations include reliance on administrative coding and prescription data, the absence of information on aura, migraine severity, or over-the-counter medication use, and the cross-sectional nature of the comorbidity analysis, which limits causal inference. Due to the nature of the data source, diagnosis of migraine was based on ICD-9 codes and not strictly on ICHD-3 criteria. Any misclassification related to reliance on administrative coding is likely to result in underestimation rather than overestimation of migraine prevalence and associated burden. Data on the use of Onabotulinum toxin A, an important preventive therapy, was unavailable.

## Conclusion

This study provides the first large national-level epidemiological assessment of migraine in Israel. Migraine in Israel is common but underdiagnosed, highly comorbid, and undertreated. Preventive use remains low, despite the availability of universal healthcare and effective therapies, suggesting that financial access is not the primary barrier. These findings emphasize the need for improved migraine recognition, integrated multidisciplinary care, and policy-level strategies to reduce the burden of this disabling condition.

## Data Availability

The raw data supporting the conclusions of this article will be made available by the authors, without undue reservation.
