# Supplementary material for: Migraine epidemiology, comorbidities and therapeutic landscape: a national population-based study
Source: Front Neurol. 2026 Jan 26;17:1743203. doi: 10.3389/fneur.2026.1743203 (PMC12883398; doi:10.3389/fneur.2026.1743203)
Supplement: Supplementary file 1 [file Supplementary_file_1.docx]

**Supplementary Table 1. Preventive Medications for Migraine and ATC Codes**

| **Drug Class** | **Active Ingredient (generic)** | **ATC Code(s)** |
| --- | --- | --- |
| **Tricyclic Antidepressants** | Amitriptyline | N06AA09 |
| **SNRIs** | Venlafaxine | N06AX16 |
|  | Duloxetine | N06AX21 |
| **Beta-Blockers** | Propranolol | C07AA05 |
|  | Metoprolol | C07AB02 |
| **Angiotensin Receptor Blockers** | Candesartan | C09CA06 |
| **Anticonvulsants** | Valproic acid | N03AG01 |
|  | Topiramate | N03AX11 |
| **CGRP monoclonal antibodies** | Erenumab | N02CD06 |
|  | Fremanezumab | N02CD05 |
|  | Galcanezumab | N02CD04 |
| **Gepants** | Rimegepant | N02CD03 |
|  | Atogepant | N02CD02 |

**Supplementary Table 2. Comorbidity Definitions and ICD-9 Codes**

| **Comorbidity Category** | **Condition / Subgroup** | **ICD-9 Code(s)** |
| --- | --- | --- |
| **Psychiatric Disorders** | Anxiety disorders | 300.0, 300.2, 300.3, 309.2 |
|  | Depression | 296.2–296.3, 311 |
| **Chronic Pain Syndromes** | Low back pain | 724.2, 724.5 |
|  | Fibromyalgia | 729.1 |
|  | Endometriosis | 617.xx |
| **Gastrointestinal** **disorders** | Inflammatory bowel disease (IBD) | 555.xx (Crohn’s), 556.xx (UC) |
|  | Irritable bowel syndrome (IBS) | 564.1 |
|  | Peptic ulcer disease | 531.xx–534.xx |
| **Vascular / Metabolic** **diseases** | Hypertension | 401.xx–405.xx |
|  | Dyslipidemia | 272.xx |
|  | Diabetes mellitus | 250.xx |
|  | Atrial fibrillation | 427.31 |
|  | Peripheral vascular disease | 443.xx, 440.xx |
|  | Cerebrovascular disease | 430.xx–438.xx |
| **Cardiac** **disorders** | Congestive heart failure | 428.xx |
|  | Myocardial infarction | 410.xx |
| **Musculoskeletal /Bone** | Osteoporosis | 733.0 |
| **Respiratory** **diseases** | Chronic pulmonary disease | 490–496 |
| **Neurological disorders** | Dementia | 290.xx, 294.xx, 331.xx |
| **Autoimmune/ Rheumatic** **diseases** | Connective tissue disease / Rheumatic disease | 710.xx, 714.xx, 725.xx |
| **Others** | Malignancy (any) | 140.xx–239.xx |
|  | HIV/AIDS | 042.xx, 043.xx, 044.xx |
|  | Renal diseases | 580.xx–589.xx |

**Supplementary Figure 1. Age at Migraine Diagnosis by Sex**


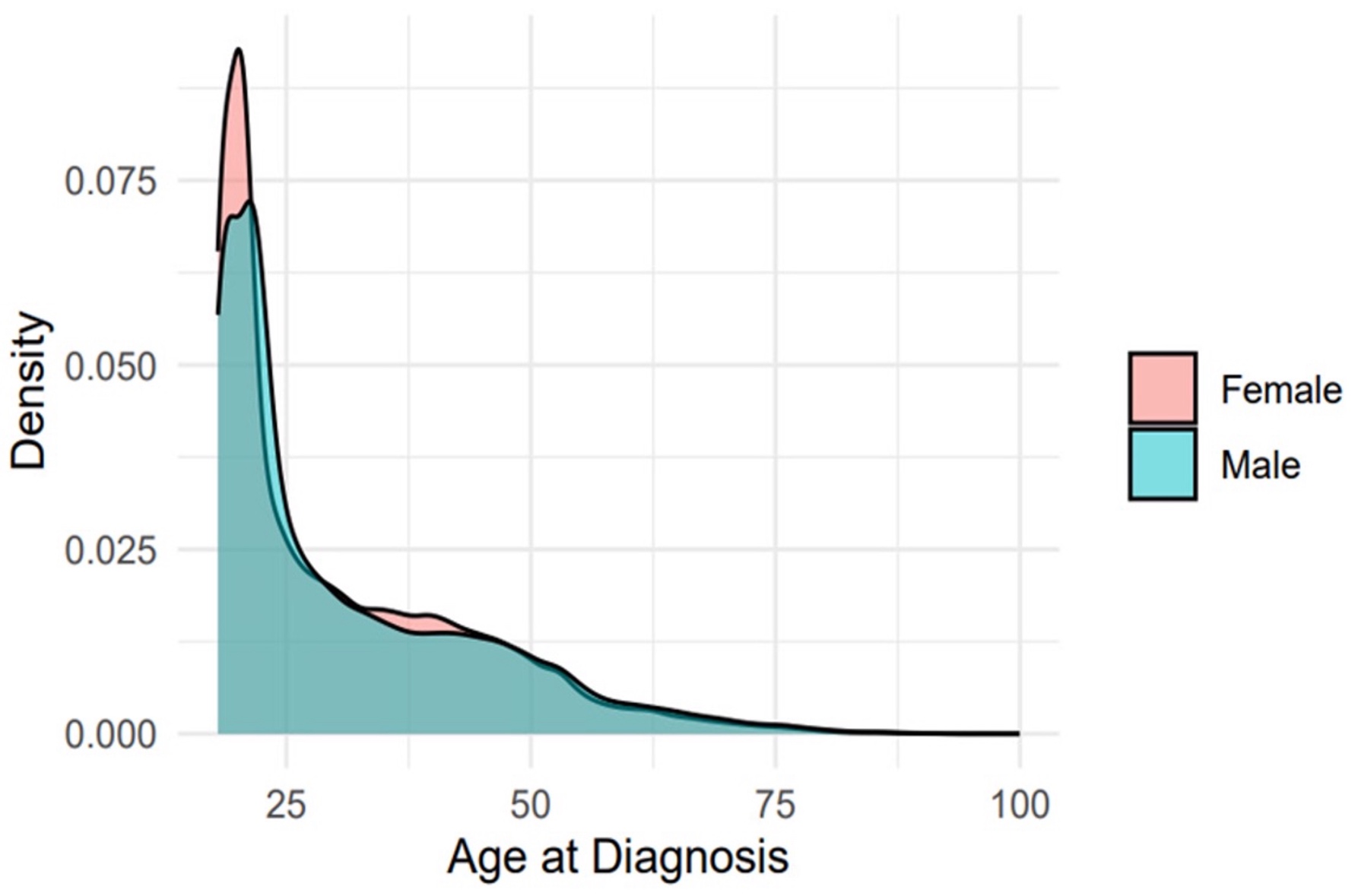


Kernel density plots of age at migraine diagnosis by sex. Density represents the relative distribution of diagnoses across age rather than absolute frequencies or probabilities at a specific age. The x-axis is restricted to ages ≥18 years. Higher density values at younger ages reflect clustering of diagnoses in early adulthood.
